# Voluntary Imbecility

**Published:** 1888-08-25

**Authors:** 


### The Doctor's Pulpit.

### VOLUNTARY IMBECILITY.

Imbecility is a long word, and has no very precise and universally accepted meaning. For the purposes of the present paper it may be defined somewhat as follows: When a man thinks less vigorously and less fairly than his best, he is suffering from an attack of imbecility. The feebler and the less just the thinking, the more pronounced the degree of imbecility. This may be called *subjective* imbecility. If the weak and unfair thinking be carried into action, the imbecility becomes *objective*. In the objective as in the subjective form, the attack may be acute or chronic, slight or severe.

In countries and at periods when there is a large, wealthy, and comparatively idle population, this disease is decidedly frequent, takes a great variety of forms, and is exceedingly difficult to cure. It is caused generally by the atrophy of certain cerebral centres from want of use, and is similar in origin and progress to that kind of atrophy of the biceps and other muscles which results from their prolonged inaction. It is curious and instructive to note the forms it takes. A case came before the writer's notice the other day. It was that of a highly-energetic man of business, whose success in life had advanced his social position very considerably. He had made the acquaintance of a few men about town, good-natured and comparatively harmless fellows, whose strong point it was that they concluded every sentence they felt called upon to utter with the elegant and convincing interrogation—"Don't cher know?" This their new associate evidently considered one of the principal diagnostic marks of social elevation and completeness. "Don't cher know" therefore concluded each of his sentences in most approved fashion. Now an imbecility like "Don't cher know" hardly sounds imbecile after a sentence which is itself an imbecility; but when it rounds off a vigorous period about practical business of unquestionable importance, it is an anti-climax which belongs to the region of the ridiculous.

Not very long ago a young fellow in the army had occasion to see a medical man. When the man of the sword approached the man of physic to shake hands he assumed a stooping posture, and, making a very obtuse angle with his elbow, brought his hand into contact with that of the doctor in a somewhat novel but decidedly characteristic fashion. For a moment the doctor thought something must be the matter with his friend's elbow-joint. But he soon perceived that there was nothing more than a very pronounced attempt to shake hands after the most approved mode. It would be curious to find out how the present mode became the mode. It is so striking and yet so amusing an imbecility that it is worth a little trouble to discover its origin. Some crooked old fellow, with no joints and a bad spinal curvature, probably did it at the outset because he could do no better. Then, perhaps, one or two profane youngsters followed him in jest. Finally, that element of imitation which coarse people call by an offensive name came into play, and so the fashion was set and has continued to the present time.

These are instances, selected because they happen to occur to the mind at the moment. The disease however takes an almost infinite variety of forms. Timidity carried to excess, and the shrinking from responsibility greatly diminish the vigour and healthiness of certain portions of the brain. A man naturally robust and self-reliant may become so timid and self-distrustful that he dare not buy himself a shilling novel at a railway station without consulting his wife or his friend. It is recorded that some of the prisoners of the Bastille, when that infamous abode of woe was destroyed, were in such a pitiable condition of imbecility that they felt unable to live the ordinary life of men among their fellow-men, and implored that they might be taken back to their cells. Dogs originally courageous have been whipped and beaten until at length the mere sight of anything like a whip or stick would make them crouch on the ground in abject terror. These latter are of course cases of involuntary imbecility, but many people from good nature or a desire to do always the "correct" thing, or a weak shrinking from standing their own ground and asserting themselves justly, have voluntarily abandoned their manhood, their intellect, and their self-reliance, and have gone over partially or entirely to the army of the imbeciles.

With women the case is even worse than with men. They are naturally of a more timid and less self-reliant disposition. Involuntarily they seek another mind to lean upon, and if the other mind be of an overbearing disposiion the yielding nature may have its individuality entirely abolished. Combined with the want of courage and self-reliance there may be, and often is, in the feminine conduct a wonderful obstinacy in following the lead of some other person or set of persons. There are women who would not dare to step over a puddle of their own judgment, who yet would jump down a lion's throat to follow fashionable leaders. What but a pronounced degree of voluntary imbecility could account for the wearing of patches on the face, of crinolines, of dress improvers, of high heels? What but an obstinacy, which is only possible to weak, unreasoning natures, could have preserved in use the fashion of dressing in a half-nude condition in midwinter, and of squeezing up the waist until respiration and digestion are alike impossible all the year round? A doctor who is worth the name is absolutely hopeless when he looks at fashionable women in the mass. Among fashionable women he includes not merely those unquestioned leaders whom everybody acknowledges to be at the summit of everything that is desirable in woman's peculiar paradise, but their imitators in the middle class, and their still more humble followers among shop-girls, factory hands, cooks, and kitchenmaids. The imbecility of fashion affects all classes of women, and the symptoms in all cases are so similar as to be almost identical.

From a medical point of view, this departure from the normal standard of brain vigour and efficiency is undoubtedly a disease. It is a variety of mental disorder, and if it were dangerous to others instead of being merely injurious to those whom it attacks, it would come under the general category of lunacy. It is necessary therefore to recognise and treat it like any other disease. The diagnosis fortunately is easy enough: the symptoms are so obvious as to be obtrusive. The cure unhappily is much more difficult. There is, however, a perfect cure, and it is to be sought on the same lines as those in which the disease originates. The imbecility we have been discussing is not like cancer or other disease over which the patient has no control. It is voluntary. The cure also must be voluntary. It consists in this and this only, that the patient must at once, and always in the future, make use of his best and honestest judgment in every circumstance of life, and firmly abide by such judgment in action. That one principle faithfully carried out will banish voluntary imbecility from the world, and in so doing will finally reverse a famous verdict of a famous philosopher, whose opinion of the brains of his fellow-men was anything but high.

